# Subclinical Myocardial Dysfunction in Patients Recovered from COVID-19 Disease: Correlation with Exercise Capacity

**DOI:** 10.3390/biology10111201

**Published:** 2021-11-18

**Authors:** Or Shimoni, Roman Korenfeld, Sorel Goland, Valery Meledin, Dan Haberman, Jacob George, Sara Shimoni

**Affiliations:** 1 The Heart Center, Kaplan Medical Center, Rehovot 7661041, Israel; sara_s@clalit.org.il (O.S.); romank2@clalit.org.il (R.K.); Sorel_g@clalit.org.il (S.G.); Valeri_M@clalit.org.il (V.M.); danha1@clalit.org.il (D.H.); Kobige@clalit.org.il (J.G.); 2 Hadassah Medical School, Hebrew University, Jerusalem 9112102, Israel; 3 Assuta Calaniot Medical Center, Ashdod 7706401, Israel

**Keywords:** myocardial strain, prolong COVID-19, exercise capacity

## Abstract

**Simple Summary:**

We examined left (LV) and right (RV) ventricular longitudinal strain in patients who had recovered from COVID-19 and assessed the correlation with exercise capacity. One hundred and eighty-four consecutive patients with history of COVID-19 disease who had been referred to rest or stress echocardiography because of symptoms, mainly dyspnea and chest pain, were included in the study. These patients were compared to 106 patients with similar age, symptoms, and risk factor profile with no history of COVID-19 disease. The patient’s age was 48 ± 12 years. Twenty-two patients had undergone severe disease. There were no differences in the LV ejection fraction and diastolic function between the groups. However, LV and RV global and free wall strain were significantly lower (in absolute numbers) in patients who had recovered form COVID-19 infection. Sixty-four patients performed exercise echocardiography. Patients with Global Longitudinal Strain (GLS) < −20% had higher exercise capacity with higher peak metabolic equivalent and exercise time compared to patients with GLS ≥ −20%. Rest and stress echocardiography in patients with symptoms after COVID-19 infection may identify patients that need further follow up to avoid long term complications of the disease. These preliminary results warrant further research, to test the natural history of these findings and the need and timing of treatment.

**Abstract:**

Aims: Myocardial abnormalities are common during COVID-19 infection and recovery. We examined left (LV) and right (RV) ventricular longitudinal strain in patients who had recovered from COVID-19 and assessed the correlation with exercise capacity. Methods and results: One hundred and eighty-four consecutive patients with history of COVID-19 disease who had been referred to rest or stress echocardiography because of symptoms, mainly dyspnea and chest pain, were included in the study. These patients were compared to 106 patients with similar age, symptoms, and risk factor profile with no history of COVID-19 disease. Clinical and echocardiographic parameters, including strain imaging, were assessed. The patient’s age was 48 ± 12 years. Twenty-two patients had undergone severe disease. There were no differences in the LV ejection fraction and diastolic function between the groups. However, LV and RV global and free wall strain were significantly lower (in absolute numbers) in patients who had recovered form COVID-19 infection (−20.41 ± 2.32 vs −19.39 ± 3.36, *p* = 0.001, −23.69 ± 3.44 vs −22.09 ± 4.20, *p* = 0.001 and −27.24 ± 4.7 vs −25.43 ± 4.93, *p* = 0.021, respectively). Global Longitudinal Strain (GLS) < −20% was present in only 37% of post COVID-19 patients. Sixty-four patients performed exercise echocardiography. Patients with GLS < −20% had higher exercise capacity with higher peak metabolic equivalent and exercise time compared to patients with GLS ≥ −20% (12.6 ± 2 vs 10 ± 2.5 METss and 8:00 ± 2:08 vs 6:24 ± 2:03 min, *p* < 0.001 and *p* = 0.003, respectively). Conclusion: In patients, who had recovered from COVID-19 infection, both LV and RV strain are significantly lower compared to control patients. The exercise capacity of these patients correlates with LV strain values. Rest and stress echocardiography in patients with symptoms after COVID-19 infection may identify patients that need further follow up to avoid long term complications of the disease. These preliminary results warrant further research, to test the natural history of these findings and the need and timing of treatment.

## 1. Introduction

Cardiovascular diseases are continuously gaining the spotlight in the discussion of Coronavirus Disease 2019 (COVID-19). Coexisting cardiovascular disease (CVD) and development of myocardial injury during hospitalization have been highly associated with mortality from COVID-19 [1], and cardiovascular complications have been marked as a major factor of mortality [2]. Moreover, evidence has shown that acute COVID-19 is associated with myocarditis, myocardial fibrosis that has led to heart failure in severe cases, acute myocardial infraction, and arrhythmias [3].

Echocardiographic tools were used as markers for higher risk of lethal outcome from COVID-19, using 2D speckle-tracking. Both Right Ventricular Longitudinal Strain (RVLS) and left ventricular Global Longitudinal Strain (GLS) were shown to have prognostic value in patients with COVID-19 and low levels were associated with higher mortality rates, hinting that COVID-19 infection might reduce systolic function [4].

Another field with rising attention in research is referred to as “post-COVID” or “long COVID”, addressing long-term COVID-19 complications. Among those complications are sustainable cardiac consequences in recovered COVID-19 patients. In one report, using cardiac magnetic resonance imaging (CMR) to evaluate the cardiac state of the said patients, no less than 78% participants had long-term abnormal cardiac findings, from which 60% suffered from myocarditis long after they recovered from COVID-19 [5]. Other CMR studies indicated similar conclusions, even if with less dramatic results. One indicated myocardial edema in the majority of the participants and late gadolinium enhancement (LGE) indicating myocardial fibrosis in about one-third of them [6], while another, made on athletes, indicated LGE in 46% of them, and myocarditis in 15% [7]. Participants in both studies had no history of CVD or cardiac involvement while infected with COVID-19.

Two-Dimensional Speckle Tracking Echocardiography findings have been shown to have prognostic value in CVD and were validated to assess ventricular function [8]. Nevertheless, while CMR findings in recovered COVID-19 patients have been discussed, STE findings remain, to date, overlooked. We hypothesized that there may be a relation between myocardial changes assessed by Speckle Tracking Echocardiography to symptoms and exercise capacity in patients recovered from COVID-19 infection. In order to shed more light on the issue of ongoing cardiac complications in recovered COVID-19 patients, we examined both the RVLS and GLS of the said patients.

## 2. Material and Methods

Consecutive patients with history of COVID-19 infection that performed ambulatory echocardiography or stress echocardiography in two centers in Israel (Kaplan Medical Center, Rehovot and Assuta Calaniot Clinic, Ashdod, Israel), between the dates 1 September 2020 and 31 January 2021, were included in the study. The patients were referred to the study by a primary physician or cardiologist. The control group included patients of similar age, gender and risk factor profile that had been referred to echocardiography for similar clinical indication at the same time period.

**Figure 1:**
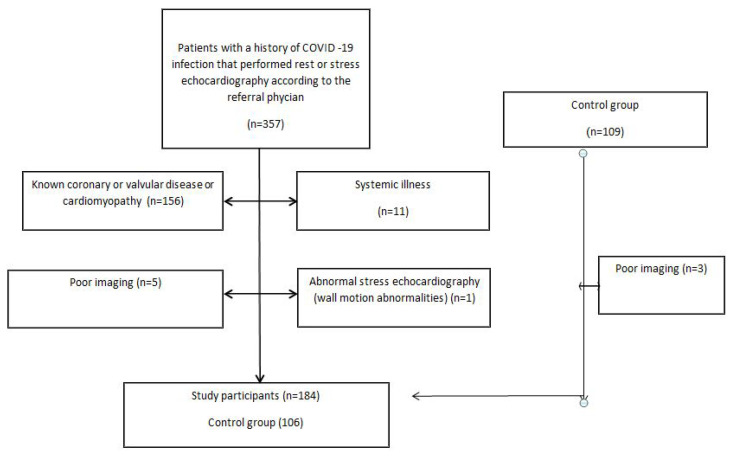
Patient flow through the study. Clinical and demographic data was reviewed including age, gender, and risk factors. Time of COVID-19 infection, disease severity, and the need for hospitalization and treatment were also collected.

Patients with known cardiac disease, including coronary artery disease, valvular disease, reduced left or right ventricular function or cardiomyopathy were excluded in both groups. Patients with history of cardiotoxic drug treatment or significant systemic disease were excluded. Patients with poor cardiac images were also excluded (Figure 1). The institutional review boards of Kaplan Medical Center (KMC 0201-20) and Assuta Medical Organization (ASMC 0003-21) approved the study.

### 2.1. Echocardiographic Measurements

Echocardiographic examination was performed using a Vivid ultrasound (E9 or E95) system (GE, Horten, Norway) or a Philips ultrasound (EPIQ 7 or CVx) system. Left ventricular (LV) and left atrial dimensions were measured in 2-D parasternal long axis view; LVEF was estimated using the bi-plane Simpson’s method; diastolic function was analysis based on mitral Doppler inflow and tissue Doppler imaging (TDI) at the lateral and septal mitral annulus, while pulmonary artery pressure was calculated by the maximal tricuspid regurgitation velocity [9,10].

### 2.2. Strain Measurements

Two-dimensional speckle-tracking strain analysis was performed offline, blinded to patient history, using Tomtec image arena version 4.6 (Tomtec, Unterschleissheim, Germany), a vendor-independent software. The left ventricular endocardial global longitudinal Strain (GLS) was obtained from the apical 4-chamber, 2-chamber, and long-axis views in an 18-segment LV model. Subsequently, longitudinal strain of all 18 LV segments was averaged to assess the GLS [11]. Global endocardial right ventricular strain and free wall strain (FWRVS) was analyzed using the same software. If more than 2 segments were inadequately tracked, we excluded the data because the images were unsuitable for speckle-tracking analysis. The cut-off values used for LV strain were based on a WASE normal values study, however we also used higher absolute values, since we wanted to see the small change in GLS and the relation to exercise [12]. The RV and FWRVS cut-off values were based on the study by Muraru et al. [13]. GLS and RV strains are usually negative numbers. However, we used absolute values: higher strain = higher absolute number (more negative) and lower strain = lower absolute number (less negative)

### 2.3. Exercise Stress Testing

In patients who had been referred to stress echocardiography, after performing a resting echocardiographic study, the patients underwent a standard treadmill exercise stress test (EST) using Bruce protocol. Subjects were questioned for symptoms every 2 min and the heart rate, blood pressure, and a 12-lead electrocardiogram were recorded at baseline, at the end of each stage, and at peak exercise. Echocardiographic imaging was performed immediately after exercise and a comparison was performed.

### 2.4. Statistical Analysis

Categorical variables were expressed as percentage and compared using Chi square or Fisher’s exact test. Continuous variables were expressed as mean ± SD and median and analyzed using T-test (for normally distributed variables) or Mann–Whitney test (for non-normally distributed variables). C statistics was performed to determine the best strain parameter related to COVID-19 infection. Initially, to understand the association between baseline clinical and echocardiographic parameters on exercise capacity, univariate analysis was performed; A regression model was utilized to simultaneously assess the influence of several variables (those which were found to be significant in the univariate analysis) on exercise capacity.

All statistical analyses were performed using commercially available software (SPSS, v22 IBM, New York, NY, USA). All tests were bilateral and *p*-value < 0.05 was considered significant.

## 3. Results

### 3.1. Patients Baseline Caracteristics and Echocardiography

The final study population included hundred eighty four consecutive patients with history of COVID-19 disease and 106 patients with similar age, gender and risk factors profile with no history of COVID-19 disease, who were referred to echocardiography study or stress echocardiography in 2 centers in Israel. As seen in Table 1, the patient’s age was 48 ± 12 years, almost half males. The number of patients with risk factors and medical treatment was relatively low. Eighty-six percent of patients had non severe disease, mainly fever, cough and muscle pain. Twenty-two patients had severe disease, including need for hospitalization, hypoxemia, and need for oxygen treatment or intubation. These patients were treated with steroids, remdesivir, and/or antibodies according to the protocols in the institutions. Troponin level was normal in all hospitalized patients, excluding one patient with troponin I level of 79 ng/mL. The CRP levels were elevated in all patients. The median time from COVID-19 infection and echocardiography was 57 (27–100) days.

The major indications for the study were dyspnea and chest pain. Other indications were palpitations and weakness. Five percent of patients were asymptomatic. The indications for the study in the control group were similar.

**Figure 2:**
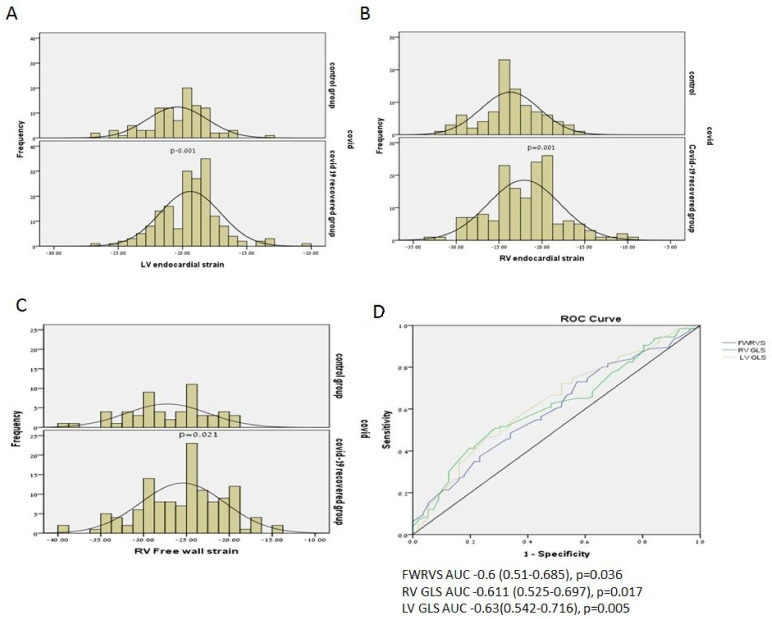
(**A**) Left GLS in COVID-19 recovered patients and controls. (**B**) Right ventricular strain in COVID-19 recovered patients and controls. (**C**) Free wall right ventricular strain in COVID-19 recovered patients and controls. (**D**) ROC for relation between LV, RV and RV free strain and history of COVID-19 infection.

The echocardiographic results of the patients and the control group are shown in Table 2. As can be seen, the echocardiographic parameters were normal and very similar in both groups. Although mild pericardial effusion was seen in eight (4.3%) patients recovered from COVID-19 compared to one patient with minimal pericardial effusion and one with mild pericardial effusion in the control group (2.8%), the difference was not statistically significant (*p* = 0.52). There were no differences in the systolic and diastolic indexes. However, the endocardial LV GLS was significantly lower (in absolute numbers) in patients recovered from COVID-19 infection (−20.41 ± 2.32% vs −19.39 ± 3.36%, *p* = 0.001, Figure 2A). GLS < −20% was present in 37% of post COVID-19 patients. Similarly, the RV global and free wall strain were significantly lower (in absolute numbers) in patients recovered from COVID-19 infection (−23.69 ± 3.44% vs −22.09 ± 4.20%, *p* = 0.001 and −27.24 ± 4.7% vs −25.43 ± 4.93%, *p* = 0.021, respectively, Figure 2B,C). ROC curves show that the best area under the curve for relation with COVID-19 history, was for LV GLS (AUC 0.63, *p* = 0.005, Figure 2D).

We compared the strain values of patients who had non severe disease to patients with severe disease. There were no significant differences in the strain values between the two groups, although the values of the RV strain and RV free wall strain were insignificantly lower in patients who had undergone severe disease (−22.16 ± 4.4 vs −20.96 ± 2.4, *p* = 0.09 and −25.66 ± 5.07 vs 23.82 ± 3.68, *p* = 0.155, respectively).The LV GLS was similar in patients that had undergone non severe disease and in patients with severe disease course (−19.44 ± 2.46 vs −19.03 ± 1.5, *p* = 0.466)

Eighty-seven (47%) patients in the COVID-19 recovered group and 52 (49%) patients in the control group were above the age of 50 years. We performed a subgroup analysis, according to patient age, and compared LV GLS, and RV strain in patients above and under 50 years in each group. In patients aged 50 years and older, there were significant differences in the values of LV GLS, RV strain and FWRV strain (−20.5 ± 2.6 vs −19.3 ± 2.6, *p* = 0.008, −23.41 ± 3.4 vs −21.46 ± 4.4, *p* = 0.011 and −27.8 ± 4.9 vs −25.47 ± 5.2, *p* = 0.039, respectively). However, in patients younger than 50 years, that have usually less respiratory and other complications, the differences in the values of LV GLS, RV strain and FWRV strain between COVID-19 recovered patients and the control group were less pronounced (−20.28 ± 2.40 vs −19.5 ± 2.14, *p* = 0.04, −24.0 ± 3.45 vs −22.7 ± 3.9, *p* = 0.072 and −26.5 ± 4.5 vs 25.4 ± 4.6, *p* = 0.3, respectively).

### 3.2. Exercise Echocardiography

Sixty-five patients with previous COVID-19 disease, had performed stress echocardiography. One patient had a positive study for ischemia and was excluded. In the remaining 64 patients, the study was negative for ischemia with no arrhythmia or adverse events. The mean age of these patients was 47 ± 11 years, 36 (56%) male. The mean exercise time was 07:06 ± 2:13 min, the peak metabolic equivalent (MET) achieved was 11.24 ± 2.8 METs and patients achieved 91.8 ± 11.68% of age predicted heart rate. Ten patients (15.6%) achieved less than 85% of age predicted heart rate, all of them due to fatigue. The mean double product was 24,996 ± 3937.

Of the 64 patients that performed stress echocardiography, GLS < −20% was seen in 20 patients and GLS > −20% in 44 patients. There were no differences between patients with GLS higher and lower than −20% with regard to age, gender, and risk factors. Patients with GLS < −20% had higher Mitral E velocity and higher E’ lateral velocity compared to patients with GLS > −20% (82 ± 12 cm/s vs 71 ± 18 cm/s and 14.7 ± 3 cm/s vs 11.7 ± 2.5 cm/s, *p* = 0.014 and *p* = 0.032, respectively). No other differences were observed in baseline echocardiographic parameters.

**Figure 3:**
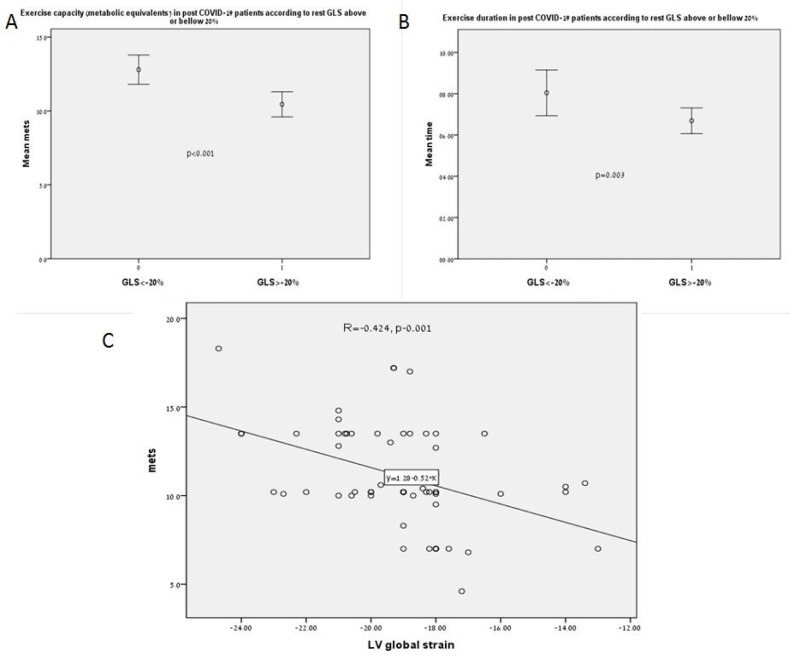
(**A**) Exercise capacity (metabolic equivalents) in post COVID-19 patients according to rest GLS above or under −20%. (**B**) Exercise duration in post COVID-19 patients according to rest GLS above or under −20%. (**C**) Correlation between LV strain and exercise capacity.

Patients with GLS < −20% had higher exercise capacity with higher peak metabolic equivalent and exercise time compared to patients with GLS ≥ −20% (12.6 ± 2 vs 10 ± 2.5 METs and 8:00 ± 2:08 vs 6:24 ± 2:03 min, *p* < 0.001 and *p* = 0.003, respectively, Figure 3A,B).

Although the exercise capacity measured in metabolic equivalents in most patients was quite good, it was related to age (r = −313, *p* = 0.012), LVEF (r = 0.292, *p* = 0.02), LVEDV (r = 0.405, *p* = 0.009), and LV GLS (r = −0.424, *p* = 0.001, Figure 3C). In the regression model only GLS and LVEDV were related to exercise capacity in COVID-19 recovered patients (*p* = 0.008 and *p* = 0.024, respectively).

No correlation was observed between RV strain and FWRVS and exercise capacity (*p* = 0.32 and 0.8, respectively).

## 4. Discussion

The main findings of our study are as follows: (1) in patients, recovered from COVID-19, the LV and RV strain is significantly lower compared to control patients with similar symptoms, age, and risk factors. This was more significant in relatively higher age. (2) In post COVID-19 patients, the subclinical impairment in LV function is related to lower exercise capacity and duration.

Various manifestations of cardiac disease have been described in patients with COVID-19 infection, including myocardial injury. Elevated troponin levels are frequent in patients with COVID-19, mainly in significantly ill patients or patients with chronic cardiovascular disease and is associated with poor prognosis [14,15]. The possible mechanisms of myocardial injury in COVID-19, include systemic hypoxia; ischemic injury, intravascular thrombosis, endothelitis, and focal necrosis; myocarditis; systemic inflammatory response syndrome; stress cardiomyopathy and right heart strain [16,17,18].

Echocardiographic abnormalities have been seen in a substantial proportion of patients during acute COVID-19 illnesses [19]. The cardiovascular abnormalities were reported in patients with severe disease and with mild disease as well [20,21,22]. Abnormal LV and RV strain during COVID-19 infection have been widely reported [4,21,22,23,24]. Abnormal LV and RV strain are also associated with poor prognosis [24,25,26,27].

Recent reports describe prolonged COVID-19 symptoms or post-COVID conditions, including shortness of breath, chest pain and fatigue, that can happen to anyone who underwent COVID-19 infection, even if the illness was mild, or no symptoms during the acute illness [28]. The cause of this and other symptoms are not known. The possible mechanisms include direct viral invasion, down-regulation of ACE2, and inflammation and immunologic response affecting the myocardium, pericardium, and conduction system [28]. Recovered patients may have persistently increased cardio metabolic demand, reduced cardiac reserve, myocardial fibrosis or scarring, and dysregulation of the renin–angiotensin–aldosterone system.

In the past months, several cardiac imaging studies, mainly CMR, showed various rates of myocardial changes in patients who had undergone COVID-19 infection. The majority of studies suggest that abnormalities of myocardial tissue characterized by CMR are common during COVID-19 recovery. The abnormalities include myocardial inflammation, ischemia, scarring, and pericardial involvement, with changes in global native T1 and T2, ECV, and LGE [6]. The studies show myocardial changes in patients who had undergone severe disease as well as patients with mild disease [5,29,30].

We found a significant difference in LV and RV strain values between patients with COVID 19 and the control group. Since there are different strain analysis software and also variability in strain values we used a large cohort of patients and controls and compared the values between the groups. Ozer et al. assessed GLS by echocardiography in 74 patients one month after COVID-19 infection, 28 of them had evidence of myocardial injury during acute illness. The strain analysis was done by different software. The LV-GLS was abnormal in 37.8% of patients [31], the rate was higher in patients with myocardial injury. The patients in this study were older with higher rate of risk factors. In addition, the age was higher and risk factors were more frequent in patients with history of myocardial injury. Although we did not find a difference in strain values between patients with severe disease and non-severe disease, we did see a more prominent strain value difference in patients of older age. In addition, the study by Ozer et al. was done a month after acute disease and as part of a routine follow-up and not symptom driven. Recently, two important reports by Lassen et al. and Baruch et al. showed no significant improvement in LV GLS in patients after COVID-19, moreover, in some patients there was a deterioration in LV GLS [32,33]. The mechanism for acute and persisting subclinical LV dysfunction is unknown, however MRI findings suggest adverse remodeling after myocarditis that may be detected by strain imaging. Another possible mechanism is endothelial and vascular dysfunction that causes subclinical LV function impairment that persists after acute COVID 19 [34].

Nuzzi et al. assessed pulmonary artery pressure and RV strain in 53 patients recovered from severe COVID-19 disease [35]. The patients were older with higher rate of risk factors, compared to patients in our study. The RV function was normal, however, the RV strain values were lower compared to our results. The RV strain values in the study by Nazzi et al. were lower compared to other studies assessing RV strain during acute COVID-19 illness [27], showing the variability in the values. In a recent study by Hayama et al., bi ventricular strain assessment was performed in patients recovered from COVID-19. The study population was very similar to patients in our study regarding patients’ age, acute illness severity, and time from disease to study. Their study investigated strain values based on troponin level. We did not assess troponin levels but the findings of abnormal myocardial deformation in patients recovered from COVID-19 is in line with this and the other studies. The findings suggest a myocardial injury may persist after the COVID-19 infection. We and others assessed longitudinal strain only, which may be less sensitive to assess myocardial injury in epicardial layers as seen in myocarditis; however, it is more accurate and reproducible compared to radial or circumferential strain. In addition, studies in myocarditis suggest impairment in longitudinal strain even when the inflammatory process is sub-epicardial [36,37].

Patients report exercise intolerance after COVID-19 infection. We found a correlation between LV strain and exercise capacity. To the best of our knowledge, no study has assessed this relation in patients recovered from COVID-19 infection. Raman et al. assessed prospectively the medium-term effect of COVID-19 infection on various organs and exercise capacity [38]. They found that 2–3 months after hospital discharge a significant proportion of patients reported breathlessness, fatigue, depression, and had limited exercise capacity. Twenty six percent of patients had cardiac MRI abnormalities and there was a correlation between native T1 and inflammatory biomarkers. The exercise capacity was lower in patients post COVID, including lower peak oxygen uptake and the capacity was worse in patients with pulmonary abnormalities. There was a correlation between six-minute walk distance and inflammatory markers. Baratto et al. assessed exercise pathophysiology in 18 patients hospitalized for COVID-19 infection, before discharge [39]. They found significantly reduced function capacity in these patients; however, this limitation was mainly related to peripheral factors such as anemia and oxygen extraction. The patients had elevated cardiac output that the authors related to an inflammatory condition. Approximately one third of the patients included, after excluding patients with low LVEF and other cardiac abnormalities, had abnormal LV and RV strain. Muscle deconditioning was a significant factor in exercise capacity in patients with severe disease that had been inactive for a period of time [40]. The patients in our study performed the exercise study at least two months after recovery and the majority had mild disease. The patients did not have deconditioning. The assessed stroke volume did not differ from patients without COVID-19. We found an independent correlation of exercise capacity with LV volume and LV strain. Both factors have been shown to be correlated with exercise capacity [41,42,43]. So, it appears that myocardial factors do correlate with exercise capacity in these patients, along with other factors that were not studied.

Limitations: Our study has some limitations: First, we do not have data on BSA and blood pressure (in patients that performed rest study only) of the patients. In addition, since it was an ambulatory echo study, we did not perform biomarker assessment. We have only limited data on troponin levels during the disease. However, the number of hospitalized patients was relatively small, and the majority of patients had mainly fever and cough. An additional limitation is that we do not have information on the clinical and echocardiographic follow up of the patient, so we cannot know the clinical significance of our findings. Another limitation is that although we found a correlation between GLS values and exercise capacity, we cannot show a direct cause and effect relation, only a correlation. In addition, we cannot relate the study finding to patient’s symptoms.

## 5. Conclusions

In patients, who have recovered from COVID-19 infection, the LV and RV strain are significantly lower compared to control patients with similar symptoms, age, and risk factors. The subclinical impairment in LV function may have a clinical implication during exercise with lower exercise capacity and duration. Echocardiography is a fast, versatile, and important imaging modality, with no side effects and low cost. The use of rest and stress echocardiography in patients with symptoms after COVID-19 infection may identify patients that need further follow up to avoid long term complications of the disease. These preliminary results warrant further research, to test the natural history of these findings and the need and timing of treatment.

## Figures and Tables

**Table 1:** Patient baseline characteristics.

	COVID-19 Recovered n = 184	Control n = 106	p
Age	48 ± 12	49 ± 13	0.39
Gender (male)	87 (47%)	52 (49%)	0.83
Hypertension	17 (9)	16 (15)	0.2
Diabetes mellitus	10 (5)	3 (3)	0.581
Hyperlipidemia	21 (11)	12 (11)	0.877
Smoking	8 (4)	2 (2)	0.44
ACEI/ARB	10 (6)	6 (6)	0.852
Beta blockers	5 (3)	4 (4)	0.88
Statins	15 (8)	9 (8)	0.9
Symptoms			
Chest pain	55 (30)	36 (34)	0.556
Dyspnea	71 (39)	41 (38)	0.880
Palpitation	28 (15)	21 (20)	0.4
Weakness	20 (11)	6 (6)	0.2
No symptoms	10 (5)	2 (2)	0.25
Disease severity			
Asymptomatic	3 (2)		
Not severe disease	157 (86)		
Severe disease	22 (12)		
Time from disease Days (25–75%)	57 (27–100)		
Laboratory results in 22 patients that were hospitalized			
Abnormal troponin	1 patient (4%)		
CRP mg/L	11.8 ± 1.3		
Hemoglobin (g/dL)	13.3 ± 1.9		
White blood cells (K/uL)	7.9 ± 0.8		
Platelets (K/uL)	175 ± 31		
Creatinine (mg/dL)	0.88 ± 0.15		
LDH U/L	378 ± 39		

ACEI—angiotensin converting enzyme inhibitor. ARB—angiotensin receptor blocker. CRP—c reactive protein LDH—lactate dehydrogenase.

**Table 2:** Echocardiography parameters of COVID-19 recovered patients and control group.

	COVID-19 Recovered Group n = 184	Control Group n = 106	*p*
Heart rate (bpm)	72.86 ± 12.26	70.63 ± 9.83	0.134
LVDD (cm)	4.73 ± 2.38	4.51 ± 36	0.235
LVSD (cm)	2.74 ± 45	2.73 ± 37	0.854
Septum thickness (cm)	1.01 ± 15	1.09 ± 88	0.195
Posterior wall thickness (cm)	0.92 ± 14	0.91 ± 14	0.475
LVEDV (mL)	103.85 ± 27.50	103.78 ± 26.78	0.99
LVESV (mL)	45.53 ± 16.70	44.14 ± 18.43	0.73
LVEF (%)	57.89 ± 2.73	58.02 ± 2.10	0.65
LVOT VTI (cm)	20.80 ± 3.52	20.57 ± 3.17	0.58
Stroke volume (mL)	71.05 ± 15.49	70.28 ± 16.35	0.71
Left atrial area (cm^2^)	16.54 ± 3.59	16.25 ± 2.96	0.49
Mitral inflow E (cm/s)	74.30 ± 17.54	74.64 ± 14.97	0.869
Deceleration time (s)	206.73 ± 69.35	202.13 ± 62.46	0.578
Mitral inflow A (cm/s)	66.61 ± 22.45	64.76 ± 17.57	0.625
E’ lateral (cm/s)	12.18 ± 4.11	11.89 ± 3.04	0.62
A’ lateral (cm/s)	9.76 ± 2.96	9.54 ± 2.71	0.62
S’ lateral (cm/s)	8.29 ± 1.65	7.88 ± 0.96	0.09
E’ septal (cm/s)	9.15 ± 2.82	9.04 ± 2.22	0.79
A’ septal (cm/s)	9.16 ± 2.07	8.96 ± 1.97	0.55
S’ septal (cm/s)	7.53 ± 1.30	7.39 ± 1.00	0.51
Right ventricular diameter (mm)	31.53 ± 4.97	32.65 ± 5.10	0.128
TAPSE (mm)	19.57 ± 2.41	20.00 ± 1.88	0.48
TR velocity (m/s)	2.42 ± 1.62	2.29 ± 0.28	0.48
S’ RV (cm/s)	11.71 ± 1.39	12.40 ± 0.84	0.16
MR/No	56	36	0.624
Mild	126	70	
Mild to moderate	2	0	
AR/No	167	95	0.43
Mild	17	11	
TR/No	59	25	0.71
Mild	124	81	
Mild to moderate	1	0	
Pericardial/No effusion	176	103	0.52
Minimal	8	2	
Mild	0	1	
FWRV strain (%)	−25.43 ± 4.93	−27.24 ± 4.7	0.021
FWRV < −23%	140 (85%)	94 (89%)	0.015
RV strain (%)	−22.09 ± 4.20	−23.69 ± 3.44	0.001
RV strain < −20.3	135 (82%)	92 (87%)	0.015
GLS (%)	−19.39 ± 3.36	−20.41 ± 2.32	0.001
GLS < −18%	130 (79%)	92 (87%)	0.006
GLS < −20%	61 (37%)	53 (50%)	0.017

LVDD—left ventricular end diastolic diameter; LVSD—left ventricular end systolic diameter; LVEDV—left ventricular end diastolic volume, LVESV—left ventricular end systolic volume; LVEF—left ventricular ejection fraction; TAPSE—tricuspid annular plane systolic excursion; TR—tricuspid regurgitation; MR—mitral regurgitation; AR—aortic regurgitation; RV—right ventricle; FW—free wall; GLS–global longitudinal strain.

## Data Availability

Data will be available on demand.
